# A comparative analysis of the amount of fluoride release, recharge and re-release after uptake in three light-cure orthodontic bonding adhesives – An in-vitro study

**DOI:** 10.12688/f1000research.155347.1

**Published:** 2024-10-04

**Authors:** Akankshya Panda, Ritesh Singla, Nishu Singla, Madhumitha Natarajan

**Affiliations:** 1 Department of Orthodontics and Dentofacial Orthopaedics, Manipal College of Dental Sciences, Manipal Academy of Higher Education, Manipal, Karnataka, 576104, India; 2 Department of Public Health Dentistry, Manipal College of Dental Sciences, Manipal Academy of Higher Education, Manipal, Karnataka, 576104, India

**Keywords:** Fluoride releasing, Orthodontic adhesives, Transbond Plus Color change, Waldent Orthobond LC, Koden EZ Bond, Fluoride recharge

## Abstract

**Background:**

Fluoride-releasing adhesives in orthodontics help protect enamel. The study compared the fluoride release, recharge, and re-release properties of three light-cure orthodontic bonding adhesives: Group A: Transbond ^TM^ Plus Color Change, Group B: Waldent Orthobond LC, and Group C: Koden EZ Bond.

**Methods:**

The study conducted an in-vitro investigation using 24 maxillary first premolar teeth bonded with one of three fluoride-releasing adhesives after etching. Each sample was placed in artificial saliva, and fluoride release was measured over 60 days using a fluoride electrode. After initial release measurement, specimens were soaked in 1000 ppm fluoride solution for 5 minutes, rinsed, and placed in new containers with distilled water before re-release measurements. Statistical Analysis: One-way ANOVA and Repeated Measures ANOVA tests were used to determine significance, with the Bonferroni posthoc test for further analysis.

**Results:**

The fluoride release rate decreased gradually over time for all three groups. The highest fluoride release occurred on Day 1 for all three bonding agents, with a significantly reduced fluoride release by Day 2. Group A exhibited consistent and highest overall fluoride release throughout the 60 days. Group B gradually declined to release fluoride until Day 7, releasing the least fluoride throughout the study. Group C had a higher fluoride release on Day 1 and Day 2 but slowly declined until Day 14. Group B and Group C sharply declined fluoride release by Day 30 and almost negligible amounts on Day 60. All three agents noted enhanced fluoride release post-recharge, with continuous release until day 14. Greater release on days 7 and 14 compared to the initial release in all three groups.

**Conclusion:**

Transbond Plus Color Change: Consistent high fluoride was released initially and post-recharge. Waldent Orthobond LC: Lower release throughout the study. Koden EZ Bond: High initial release and post-recharge but continuing to decrease until Day 14.

## Introduction

Orthodontic treatments, which correct dental misalignments, pose challenges for maintaining oral hygiene and preventing dental caries. ^1^^,^^2^ The fluoride-releasing bonding adhesives are crucial in strengthening the enamel, making it more resistant to acid attacks and reducing caries risk. ^3^^,^^4^ Fluoride released by the adhesives helps remineralization, allowing the tooth’s decalcified enamel near the brackets to repair itself to some extent by forming fluorapatite crystals that strengthen enamel after being demineralized by acids. ^5^^–^^7^ It disrupts bacterial adhesion and colonization, interferes with sugar metabolism by bacteria, and thus reduces acid production, preventing demineralization and caries formation. ^8^ It also helps arrest or reverse incipient carious lesions of enamel. ^9^ The ability of adhesives to recharge fluoride ions from the oral environment to replace lost fluoride and re-release fluoride is essential for ongoing protection. After the initial release and uptake, the fluoride within the adhesive can continue to be released gradually over time, providing a sustained protective effect. ^10^^–^^12^

The mechanisms of releasing, absorbing, and re-releasing fluoride through adhesive bonding are complex and influenced by various factors. Factors such as adhesive composition, filler materials, curing techniques, and environmental conditions influence fluoride release and uptake. ^13^^–^^16^ Adhesives with higher concentrations of fluoride-releasing compounds typically offer more protection by releasing higher amounts and longer durations of fluoride. The type, size, and distribution of fillers can also impact the adhesive’s physical properties, including its porosity and the rate at which fluoride is released. ^17^^,^^18^ The overall effectiveness of the adhesive in preventing caries can depend on how well the adhesive bonds to the tooth and how well it maintains fluoride release throughout treatment. Understanding how bonding adhesives release, absorb, and re-release fluoride is crucial for orthodontists to choose the right products and implement strategies that enhance both the effectiveness of the orthodontic treatment and prevent caries during orthodontic treatment.

This study was planned to explore different adhesive formulations under experimental conditions to understand the dynamics of fluoride release and its implications for caries prevention. The study investigated the fluoride release, uptake, and re-release behaviors of three commercially available light-cure orthodontic bonding adhesives: Transbond Plus Color Change, Waldent Orthobond LC, and Koden EZ Bond using McLaughlin Bennett 5.0 brackets by Forestadent. It included in-vitro experiments simulating clinical scenarios to monitor the fluoride release rate from each adhesive over time. The findings aim to compare the three adhesives to see if one offers superior performance regarding fluoride release, uptake, and re-release. These behaviors can significantly impact the effectiveness of caries prevention in orthodontic treatments. The research could improve oral health outcomes for orthodontic patients and provide insights for clinicians and researchers in orthodontics and preventive dentistry.

## Methods

This was an in-vitro experiment conducted on 24 extracted first premolar teeth. The research’s permission was acquired from the Ethics Committee of Kasturba Medical College and Kasturba Hospital (IEC-51/2022). Based on a previous study, the sample size of 24 premolar teeth (8 in each group; 3 groups) was determined using power analysis. ^13^ The sample collection process involved obtaining anonymous first premolar teeth from the Department of Oral Surgery, Manipal College of Dental Sciences, Manipal. These teeth were extracted from orthodontic patients who needed these extractions as part of their treatment. Due to the in-vitro nature of the study involving experimentation on anonymized teeth samples, patient consent was waived by the ethical committee. ^19^ Upper first premolars were chosen due to their prevalence in orthodontic extractions, suitability for bracket bonding, and susceptibility to demineralization during orthodontic treatment. The inclusion criteria for the samples were sound crown structure, free of caries and cracks, no developmental defects, and absence of restorations. After extraction, the teeth were carefully handled to avoid damage or contamination. They were cleaned using non-fluoridated pumice and stored in distilled water to prevent dehydration until use. Each tooth was mounted to the cementoenamel junction in cold cure acrylic using a PVC pipe template (19 mm diameter, 25 mm height).

Before bonding, the buccal surfaces of the upper first premolar teeth were etched with 37% phosphoric acid for 30 seconds to enhance bond strength. Three light cure orthodontic bonding agents were used: Group A: Transbond Plus Color Change, Group B: Waldent Orthobond LC, and Group C: Koden EZ Bond. Brackets (McLaughlin Bennett 5.0 by Forestadent) were positioned and bonded according to the manufacturer’s guidelines for each agent. A thin layer of bonding agent was applied to the bracket base, and the bracket was pressed onto the tooth. Excess adhesive was removed. The brackets were cured using a 3M S10 ELIPAR Light Curing Unit set at 1200 mW/cm ^2^, with 10 seconds of light exposure from the incisal edge and another 10 seconds from the gingival, mesial, or distal edges. The light was positioned close to the bracket base to ensure proper polymerization. Each bracket bonding procedure followed the same standardized protocol to ensure consistency and comparability between the different bonding agents used in the study.

After bonding the brackets to etched enamel surfaces, specimens were placed in plastic vials with 5 ml of artificial saliva containing carboxymethyl cellulose to simulate the oral environment. Fluoride release was measured to assess the amount of fluoride ions released from the orthodontic adhesive over time. Fluoride concentration was recorded using an Orion fluoride ion meter with a selective electrode. The electrode, featuring a lanthanum fluoride crystal, measures the potential differences fluoride ions create. To ensure accurate measurements, TISAB was used to provide a uniform ionic strength, adjust pH, and break up fluoride complexes.

Samples and standard solutions were prepared in 100 ml beakers with equal amounts of TISAB, maintaining a total volume of 30 ml. The meter was calibrated using standard fluoride solutions of 0.1 ppm, 1 ppm, and 10 ppm. Initial fluoride release was measured by immersing the electrodes for 3 minutes on days 1, 2, 3, 4, 5, 6, 7, 14, 30, and 60 post-bonding. These measurements are crucial for evaluating the fluoride-releasing capabilities of different orthodontic bonding agents and their potential benefits for caries prevention and enamel remineralization. By monitoring fluoride release over specific intervals, the study aims to determine the efficacy and duration of fluoride release, which is essential for assessing the clinical implications of the bonding agents used in orthodontic treatment.

After the initial fluoride release measurements, the specimens undergo fluoride uptake and rerelease to simulate dynamic oral environment exposure. **Fluoride Uptake**: Specimens were soaked for 5 minutes in a 1000 ppm fluoride solution, rinsed with distilled water, dried, and transferred to new containers with 5 ml of distilled water each time before recording post-recharge measurements. **Fluoride Rerelease**: Fluoride concentration is measured using a fluoride meter on days 1, 2, 7, 14, 30, and 60 post-immersion. These measurements assess the adhesive’s fluoride uptake capacity, retention, and rerelease patterns, providing insights into their sustained fluoride-releasing potential. This helps evaluate their effectiveness in replenishing fluoride ions, promoting caries prevention, and enamel remineralization during orthodontic treatment.

### Statistical analysis

The study data was analyzed using the statistical package SPSS 26.0 (SPSS Inc., Chicago, IL). Descriptive statistics was performed to assess the mean and standard deviation of the respective groups. The data’s normality was evaluated using the Shapiro-Wilk test, indicating a normal distribution (P>0.05), allowing for parametric tests. The mean difference between the groups was determined using the ‘One-way ANOVA test’ and within-group analysis by ‘repeated measures of ANOVA test’ followed by the ‘Bonferroni posthoc test.’ The level of significance was set at P<0.05.

## Results

**Figure 1.**
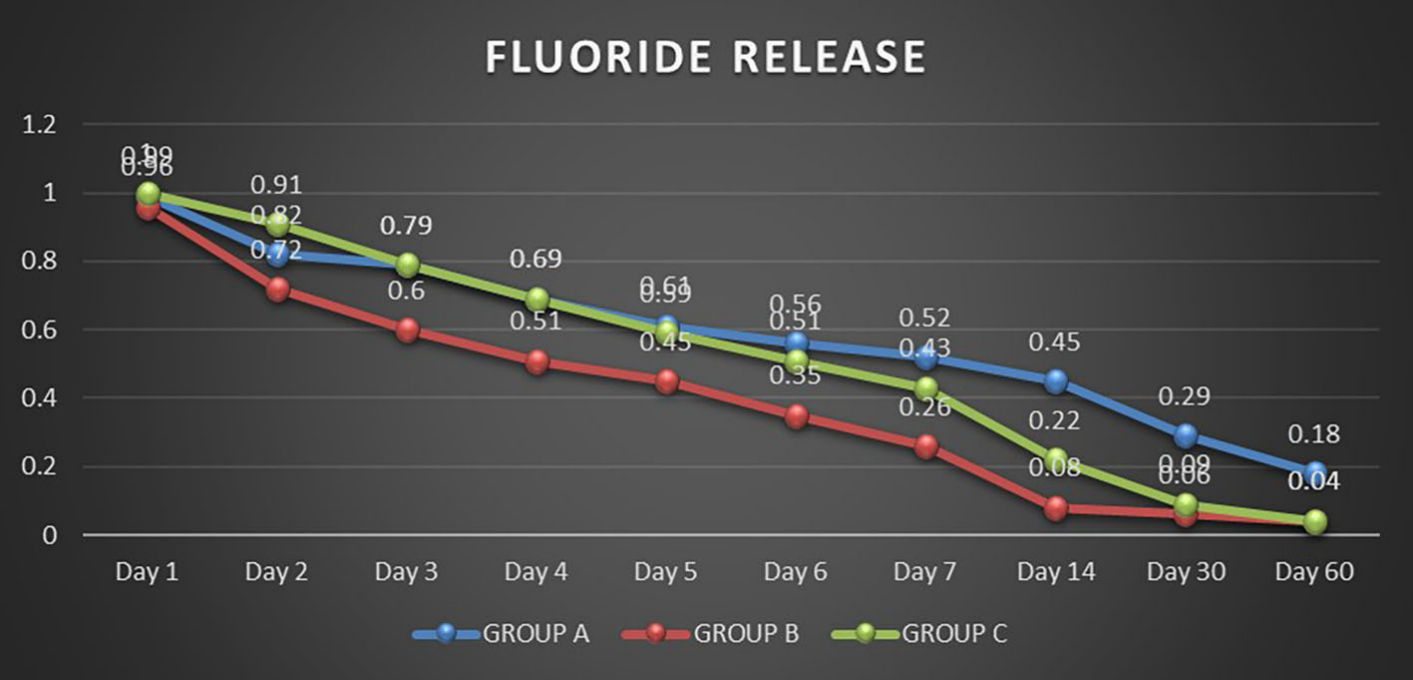
Comparison of initial fluoride release in the groups'.

Fluoride released by three light-cured bonding agents (Group A: Transbond Plus Color Change, Group B: Waldent Orthobond LC, Group C: Koden EZ Bond) was analyzed over 60 days using an ion-selective electrode. Repeated Measures ANOVA showed significant differences within each group over time (P<0.05). There was a significant decrease in the release of fluoride levels between all pair days except for Day 2 vs Day 3 in Group A (P>0.05). It was observed that the fluoride release rate decreased gradually for all three groups over time ( Figure 1). The highest fluoride release occurred on Day 1 for all three bonding agents, with a significantly decreased fluoride release by Day 2. Group A showed the consistent and highest overall fluoride release compared to the other two groups, gradually reducing over time. Group B gradually declined to release fluoride until Day 7, releasing the least fluoride throughout the study. Group C had a higher fluoride release on Day 1 and Day 2 but continued to decrease until Day 14 (Table S1, Extended data). Group B and Group C sharply declined fluoride release by Day 30 and almost negligible amounts on Day 60.

On day 1, all three groups exhibited similar fluoride release, with Group C slightly higher and no statistically significant differences. On day 2, Group C exhibited significantly higher fluoride release than Group A and Group B (P<0.0001). From day 3 to day 6, Groups C and A showed similar levels of fluoride release. However, from day 7 onwards, Group A released significantly higher levels of fluoride than Group C. Additionally, starting from day 2, Group A consistently exhibited significantly higher fluoride release than Group B. Group C also demonstrated a significantly higher fluoride release than Group B except on day 60, when there was no significant difference between them (Table S2, Extended data).

**Figure 2.**
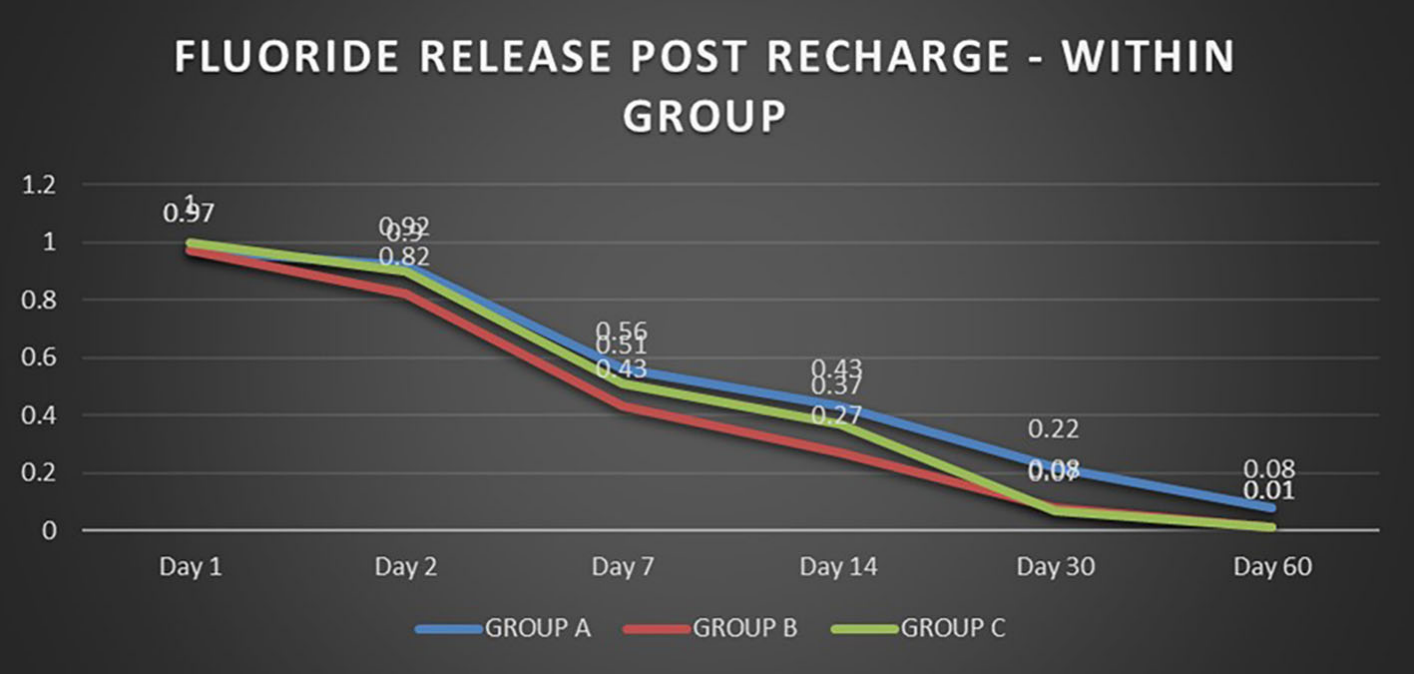
Comparison of fluoride release post recharge in the groups.

**Figure 3.**
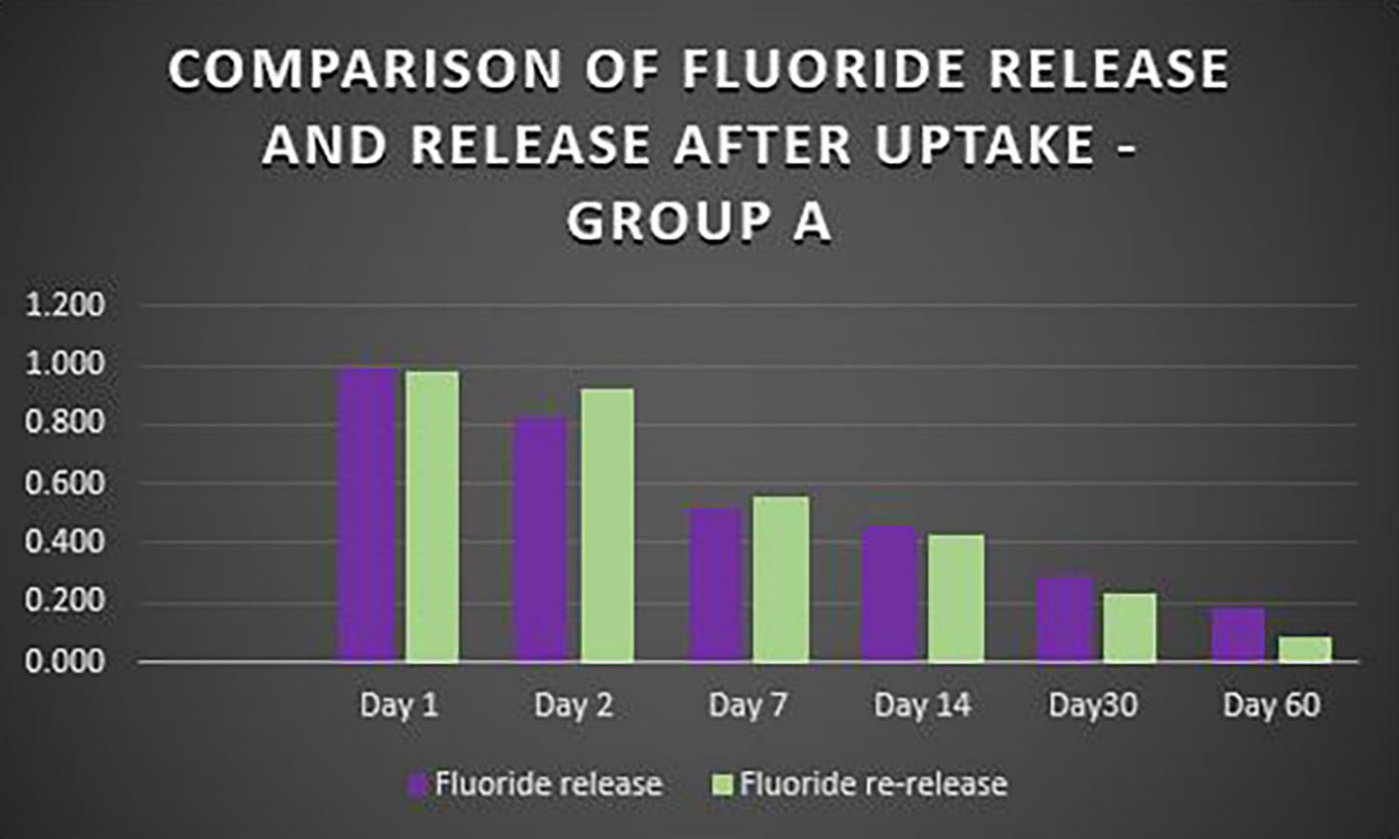
Comparison of fluoride release and release after uptake - Transbond Plus Colour Change (Group A).

**Figure 4.**
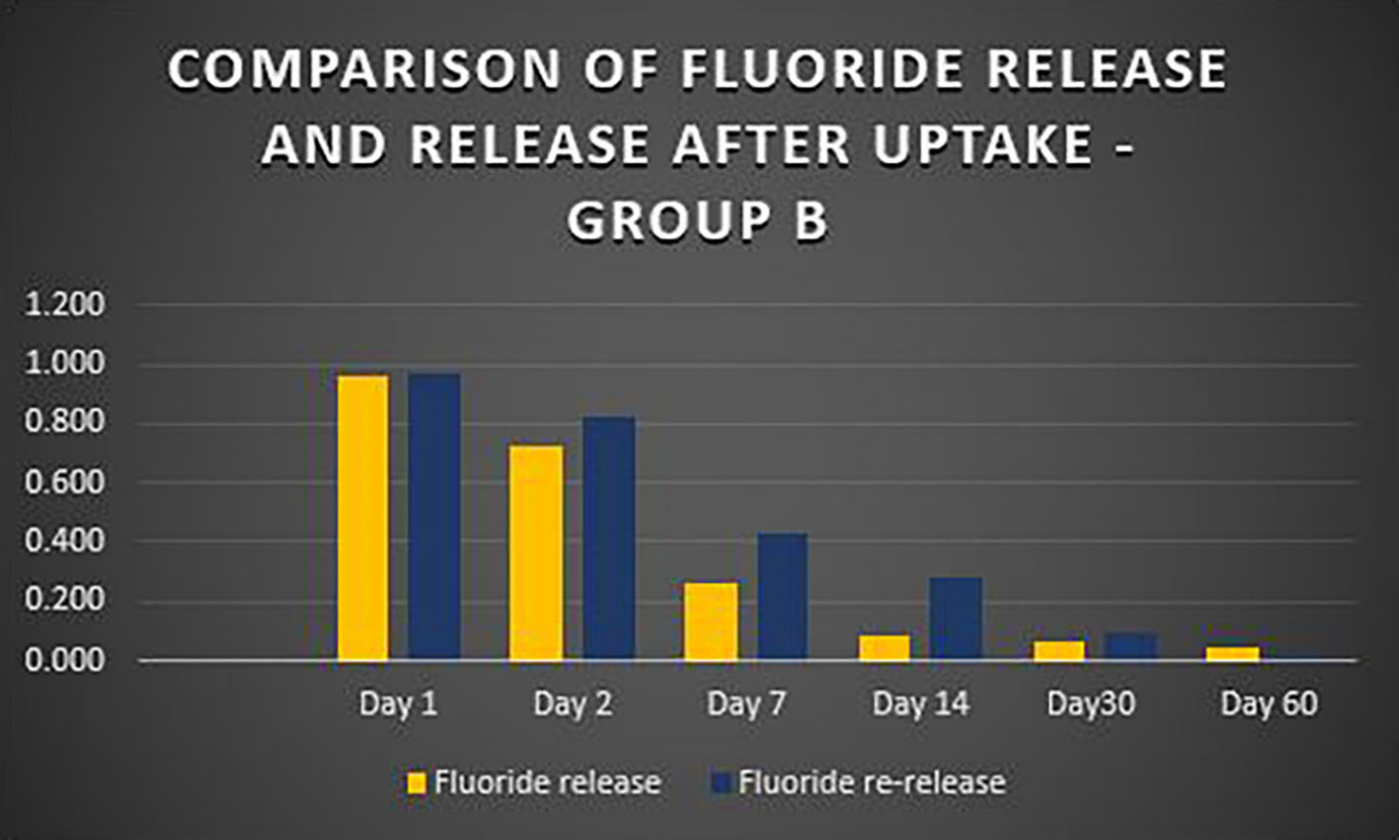
Comparison of fluoride release and release after uptake - Waldent Orthobond LC (Group B).

**Figure 5.**
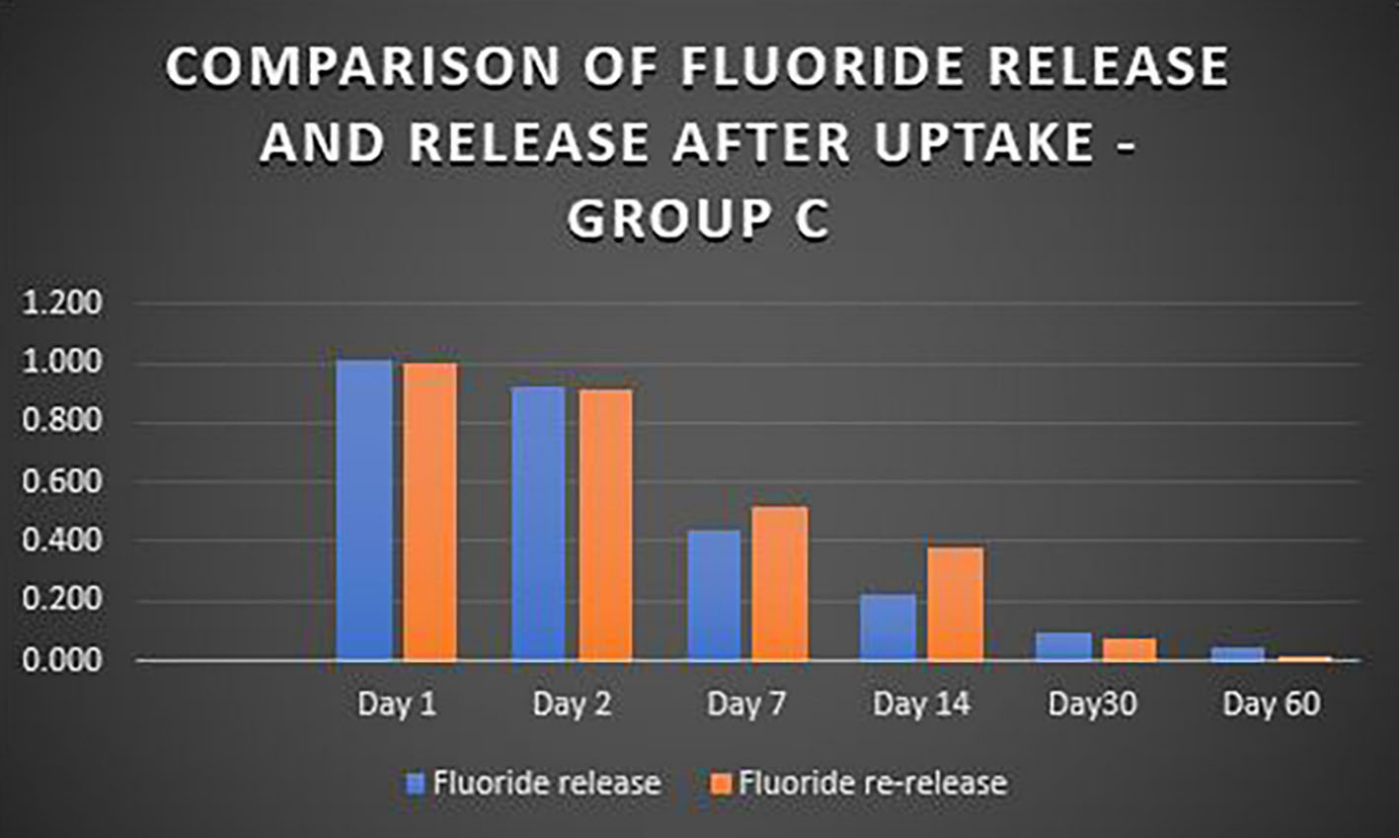
Comparison of fluoride release and release after uptake - Koden EZ bond (Group C).

When the three orthodontic adhesives were recharged with a 1000 ppm NaF solution, the rate of fluoride re-release declined over time, similar to the initial post-bonding release ( Figure 2). However, fluoride was increased in all three agents post-recharge compared to the initial release, with the sustained release until Day 14 ( Figures 3, 4, and 5). It was observed that Days 7 and 14 showed higher release in all groups after recharging compared to the initial release. Repeated Measures ANOVA showed a significant decrease in fluoride re-release within each group over time (P<0.05) ( Table 1). Comparison between the groups revealed that on Day 1, Group C exhibited slightly higher fluoride release than Groups A and B. After that, starting from Day 2, Group A released higher fluoride than the other two groups (Group B and C). On Day 30 and Day 60, Groups B and C had negligible release; Group A had significant release throughout 60 days post-recharge ( Table 2).

**Table 1.** Comparison of fluoride release after recharge - within group.

		Group A	Group B	Group C
Day 1		0.97±0.01	0.97±0.008	1.00±0.009
Day 2		0.92±0.02	0.82±0.009	0.90±0.006
Day 7		0.56±0.02	0.43±0.01	0.51±0.01
Day 14		0.43±0.01	0.27±0.009	0.37±0.005
Day 30		0.22±0.03	0.08±0.005	0.07±0.006
Day 60		0.08±0.01	0.01±0.006	0.01±0.005
Bonferroni posthoc test	Day 1 vs. Day 2	*0.0001	*0.0001	*0.0001
	Day 1 vs. Day 7	*0.0001	*0.0001	*0.0001
	Day 1 vs. Day 14	*0.0001	*0.0001	*0.0001
	Day 1 vs. Day 30	*0.0001	*0.0001	*0.0001
	Day 1 vs. Day 60	*0.0001	*0.0001	*0.0001
	Day 2 vs. Day 7	*0.0001	*0.0001	*0.0001
	Day 2 vs. Day 14	*0.0001	*0.0001	*0.0001
	Day 2 vs. Day 30	*0.0001	*0.0001	*0.0001
	Day 2 vs. Day 60	*0.0001	*0.0001	*0.0001
	Day 7 vs. Day 14	*0.0001	*0.0001	*0.0001
	Day 7 vs. Day 30	*0.0001	*0.0001	*0.0001
	Day 7 vs. Day 60	*0.0001	*0.0001	*0.0001
	Day 14 vs. Day 30	*0.0001	*0.0001	*0.0001
	Day 14 vs. Day 60	*0.0001	*0.0001	*0.0001
	Day 30 vs. Day 60	*0.0001	*0.0001	*0.0001

* P<0.05 is statistically significant.

**Table 2.** Comparison of fluoride release after recharge in-between group.

	Group A	Group B	Group C	P value	Post Hoc Test	P value
Day 1	0.977±0.019	0.971±0.01	1.001±0.011	0.0001 *	A vs. B	0.99
					A vs. C	0.0001 *
					B vs. C	0.0001 *
Day 2	0.927±0.023	0.826±0.010	0.909±0.007	0.0001 *	A vs. B	0.0001 *
					A vs. C	0.01 *
					B vs. C	0.0001 *
Day 7	0.567±0.027	0.432±0.014	0.517±0.013	0.0001 *	A vs. B	0.0005 *
					A vs. C	0.0001 *
					B vs. C	0.0001 *
Day 14	0.432±0.019	0.277±0.011	0.377±0.006	0.0001 *	A vs. B	0.0001 *
					A vs. C	0.0001 *
					B vs. C	0.0001 *
Day 30	0.228±0.039	0.09±0.006	0.072±0.007	0.0001 *	A vs. B	0.0001 *
					A vs. C	0.0001 *
					B vs. C	0.34
Day 60	0.085±0.011	0.017±0.007	0.012±0.006	0.0001 *	A vs. B	0.0001 *
					A vs. C	0.0001 *
					B vs. C	0.96

* P<0.05 is statistically significant (Friedman test & Bonferroni Postthoc test).

## Discussion

The emergence of white spot lesions near orthodontic brackets due to early enamel demineralization is a significant concern for orthodontists. ^1^^,^^2^ The effectiveness of standard preventive measures like sealants, topical fluorides, and oral hygiene instructions for orthodontic patients may be limited if the challenges of maintaining oral hygiene with braces are not adequately addressed. Additionally, a study by Mohammed Althagafi found that fluoride pre-treatment before bonding reduces bond strength, increasing bracket debonding and chairside time. ^20^ An ideal preventive system should deliver controlled fluoride release at plaque-prone sites around orthodontic brackets without relying on patient cooperation. Incorporating fluoride-releasing adhesives into orthodontic treatment can thus offer significant benefits in immediate bond strength and long-term enamel protection. ^21^^–^^23^

The protection against demineralization varies with the amount of fluoride released by different bonding materials. ^13^^–^^16^ The efficacy of fluoride-releasing materials is influenced by how well they release, recharge, and re-release fluoride, which is affected by the bonding agent’s matrix, size, and shape. ^17^^,^^18^ Understanding the fluoride-releasing potential of different bonding adhesives is essential for selecting the most effective materials to protect enamel during orthodontic treatment. This study compares the fluoride-releasing potential of three commercially available light-cure orthodontic bonding adhesives (Transbond Plus Color Change, Waldent Orthobond LC, Koden Ez bond).

The study revealed that on Day 1, there was a “burst effect” of fluoride release, followed by a gradual decline from Day 2. This pattern was consistent across all three adhesives. Chan DCN et al. and McNeill CJ et al. observed similar patterns in their studies, with significant drops in fluoride release after 24 hours, continuing to decrease over time. ^24^^,^^25^ Transbond Plus Color Change exhibited the consistent and highest overall fluoride release throughout the 60 days. Koden Ez bond had the highest fluoride release on Day 1 and Day 2 and a similar release as Transbond Plus Color Change till Day 6 but continued to decrease until Day 14. Waldent Orthobond LC gradually declined to release fluoride until Day 7, releasing the least fluoride throughout the study. Waldent Orthobond LC and Koden Ez bond sharply declined fluoride release by Day 30 and almost negligible amounts on Day 60.

Even though a similar comparison group wasn’t found in other studies, according to similar research, Transbond Plus Color Change Adhesive significantly reduces enamel demineralization around brackets compared to traditional adhesives. It outperforms other adhesive groups in preventing white spot lesions, such as reported by Osama et al. that Transbond Plus Color Change Adhesive was more effective than the conventional adhesive Transbond XT. ^26^ Bhushan R et al. found it more effective than the GC Fuji Ortho LC and Vitremer groups. ^27^ At the same time, Passalini P reported it more effective than Orthodontic Fill Magic. ^28^ On the contrary, in a study by Vittorio Cacciafesta et al., Fuji Ortho LC released the highest amount of fluoride. In contrast, Transbond Plus released negligible amounts of fluoride, among other bracket-bonding adhesives. ^16^

Transbond Plus Color Change adhesive contains fluoro-aluminosilicate glass, which acts as a reservoir for fluoride. The adhesive is hydrophilic, allowing it to perform effectively even in the presence of saliva. The cured, cross-linked structure of Transbond Plus Color Change is designed to be stable, allowing for the controlled release of fluoride over an extended period. As a result, fluoride is steadily released even after the initial application period. This matrix helps to retain fluoride and release it gradually, providing ongoing protection against demineralization. ^26^^,^^29^ Waldent Orthobond LC and Koden EZ Bond are relatively new materials and lack extensive documentation on fluoride release, recharge, and re-release capabilities, unlike Transbond Plus Color Change. In the current study, Waldent Orthobond LC may offer competitive bonding strength and performance but might not match the fluoride management features of Transbond Plus Color Change. Koden EZ Bond does offer high initial fluoride release and post-recharge for the first week, continuing to decrease till Day 14.

Consistent with prior research, each bonding agent showed an enhanced recharge level with each additional fluoride treatment, even after aging. ^29^^,^^30^ Similarly, the correlation between pre-recharge release levels and post-recharge re-release levels aligns with previous studies. ^30^^,^^31^ These results suggest that the rechargeability of these composites is influenced by the availability of sites within the material capable of retaining absorbed fluoride. ^31^^,^^32^ Waldent Orthobond LC had the lowest re-release throughout the study. Koden Ez bond exhibited the highest initial release post-recharge but declined sharply after day 2, showing negligible release by day 60. Transbond Plus Color Change had a lower initial release but demonstrated superior recharge and re-release properties, suggesting a greater capacity to uptake and release fluoride over time. ^26^

The current orthodontic bonding agents are mainly designed for their bonding strength and ease of use. They offered their highest fluoride ion release within the first 24 hours, followed by a low level of long-term fluoride ion release. ^3^^,^^4^ Most fluoride-releasing adhesives exhibit low release after 2 months, necessitating sustained release throughout the typical 2-year orthodontic treatment. Therefore, it is recommended to use additional fluoride sources, such as toothpaste and mouthwash, during treatment. ^10^^–^^12^ External fluoride sources replace fluoride ions in the resin matrix, effectively recharging the bonding agent. The recharge process depends on exposure concentration, duration, and resin matrix properties. ^17^^,^^18^ In this study, bonding adhesives were recharged by dipping samples in 1000 ppm sodium fluoride solution for 5 minutes, simulating daily use of fluoridated toothpaste for 6 months. Periodic re-fluoridation of bonding agents is necessary to replenish utilized fluoride ions and prevent enamel demineralization during orthodontic treatment. This study’s findings align with previous research, emphasizing the importance of continuous lower fluoride levels rather than one-time high-concentration applications. ^10^^–^^12^

### Limitations

While the current in-vitro study provides valuable preliminary insights into the performance of three bonding agents, its limitations must be considered. The controlled laboratory environment may not fully replicate the complex conditions of an oral environment, which can affect the applicability of the findings. Therefore, further research is necessary to validate and expand upon these results. Conducting in vivo studies with larger sample sizes and incorporating a wider range of bonding materials will offer a more comprehensive understanding of the most effective and practical orthodontic adhesives. Such research will help confirm the initial findings and clarify how these materials perform under real-world conditions.

## Conclusion

All three orthodontic adhesives showed a significant decrease in fluoride release after day 1, continuing through day 60. After recharging with a 1000 ppm fluoride solution, the fluoride release levels of the adhesives were similar to their initial release. Transbond Plus Color Change Adhesive demonstrated stable and sustained higher fluoride release over a 60-day study period. Although it did not have the highest initial fluoride release or the most robust recharge and re-release capabilities, its balanced combination of these properties and its color-changing feature indicate proper curing, making it the most suitable option for clinical use among the materials studied. Koden Ez Bond adhesive exhibited the highest initial and post-recharge fluoride release but sharply declined by day 30 and almost negligible amounts on Day 60. Waldent Orthobond LC adhesive consistently showed lower fluoride release initially and after recharging than the other adhesives.

### Ethics and consent

The research’s permission was acquired from the Ethics Committee of Kasturba Medical College and Kasturba Hospital (IEC no.51/2022, Dated 31-12-2022). Due to the in-vitro nature of the study involving experimentation on anonymized teeth samples, patient consent was waived by the ethical committee. ^19^

## Data Availability

Figshare: A comparative analysis of the amount of fluoride release, recharge, and re-release after uptake in three light-cure orthodontic bonding adhesives – An in-vitro study. https://doi.org/10.6084/m9.figshare.27005521.v1 ^33^ This project contains the following underlying data:
• Data.xlsx Data are available under the terms of the Creative Commons Attribution 4.0 International license (CC-BY 4.0). Figshare: A comparative analysis of the amount of fluoride release, recharge, and re-release after uptake in three light-cure orthodontic bonding adhesives – An in-vitro study. https://doi.org/10.6084/m9.figshare.27005521.v1 ^33^ This project contains the following extended data:
• Tables.pdf Data are available under the terms of the Creative Commons Attribution 4.0 International license (CC-BY 4.0).
