# Supplementary material for: A comparative analysis of the amount of fluoride release, recharge and re-release after uptake in three light-cure orthodontic bonding adhesives – An in-vitro study
Source: F1000Res. 2025 Dec 22;13:1133. Originally published 2024 Oct 4. [Version 2] doi: 10.12688/f1000research.155347.2 (PMC12780519; doi:10.12688/f1000research.155347.2)
Supplement: Peer review history

# Reviewer report: Reviewer response for version 2

**DOI:** 10.5256/f1000research.193374.r445359

**Published:** 2026-01-07

**Authors:** Nidhin Philip Jose

**Affiliations:** 1 Manipal Academy of Higher Education, Karnataka, India

The study has an acceptable methodology and is novel in its objective. It is clinically relevant given the plethora of options a clinician faces and the fact that WSL are an undesirable consequence of orthodontic treatment. It will be interesting to see further research combining fluoride release with characterization of adhesive surface and bond strength to understand things in a better perspective.

Is the work clearly and accurately presented and does it cite the current literature?

Yes

If applicable, is the statistical analysis and its interpretation appropriate?

I cannot comment. A qualified statistician is required.

Are all the source data underlying the results available to ensure full reproducibility?

Yes

Is the study design appropriate and is the work technically sound?

Yes

Are the conclusions drawn adequately supported by the results?

Yes

Are sufficient details of methods and analysis provided to allow replication by others?

Yes

Reviewer Expertise:

Othodontics, Material Research

I confirm that I have read this submission and believe that I have an appropriate level of expertise to confirm that it is of an acceptable scientific standard.

# Reviewer report: Reviewer response for version 2

**DOI:** 10.5256/f1000research.193374.r445358

**Published:** 2026-01-07

**Authors:** Sivakumar Arunachalam

**Affiliations:** 1 Clinical Oral Health Sciences, School of Dentistry, International Medical University (IMU), Kuala Lumpur, Malaysia

Thank you for the opportunity to review the manuscript tiled “A comparative analysis of the amount of fluoride release, recharge and re-release after uptake in three light-cure orthodontic bonding adhesives – An in-vitro study”. The revised version demonstrates improvement in clarity and completeness compared to earlier versions, particularly in the description of methods and interpretation of results. However, I have few more queries that may need to be addressed so that the manuscript can provide meaningful evidence for clinicians and researchers interested in preventive orthodontic materials.

1. While the fluoride release and recharge behavior of Transbond Plus Color Change has been previously studied, the inclusion of lesser documented materials (Waldent Orthobond LC and Koden EZ Bond) adds value. However, the authors should more explicitly highlight this aspect in the introduction and discussion to strengthen the manuscript’s novelty.
2. Clarify whether the calculated effect size was based on fluoride release at a specific time point (example day 1 or cumulative release) and how this aligns with the repeated-measures design?
3. Elaborate on how the observed fluoride release values might translate clinically (in relevance to enamel remineralization thresholds or prevention of white spot lesions).  
4. The use of a 1000 ppm fluoride solution for 5 minutes is reasonable and commonly reported, but the rationale for equating this protocol to “daily use of fluoridated toothpaste for six months” should be better justified or supported with a reference.
5. The figures effectively illustrate fluoride release trends. However, please add clear units (ppm) on all Y-axes and ensure consistent labelling across figures to enhance clarity.
6. Provide a brief description from Tables S1 and S2 of their key findings in the main text so that it would help readers who may not immediately consult supplementary data.
7. The terms “re-release,” “rerelease,” and “release after uptake” are used interchangeably. For clarity, the authors should standardize terminology throughout the manuscript.

Is the work clearly and accurately presented and does it cite the current literature?

Yes

If applicable, is the statistical analysis and its interpretation appropriate?

I cannot comment. A qualified statistician is required.

Are all the source data underlying the results available to ensure full reproducibility?

Yes

Is the study design appropriate and is the work technically sound?

Yes

Are the conclusions drawn adequately supported by the results?

Yes

Are sufficient details of methods and analysis provided to allow replication by others?

Yes

Reviewer Expertise:

Orthodontics, dental education

I confirm that I have read this submission and believe that I have an appropriate level of expertise to confirm that it is of an acceptable scientific standard, however I have significant reservations, as outlined above.

# Reviewer report: Reviewer response for version 2

**DOI:** 10.5256/f1000research.193374.r443977

**Published:** 2025-12-29

**Authors:** Ganugapanta Vivek Reddy

**Affiliations:** 1 Narayana Dental College, Nellore,, India

The corrections made by the author are satisfactory.

Is the work clearly and accurately presented and does it cite the current literature?

Yes

If applicable, is the statistical analysis and its interpretation appropriate?

Yes

Are all the source data underlying the results available to ensure full reproducibility?

Yes

Is the study design appropriate and is the work technically sound?

Yes

Are the conclusions drawn adequately supported by the results?

Yes

Are sufficient details of methods and analysis provided to allow replication by others?

Yes

Reviewer Expertise:

NA

I confirm that I have read this submission and believe that I have an appropriate level of expertise to confirm that it is of an acceptable scientific standard.

# Reviewer report: Reviewer response for version 1

**DOI:** 10.5256/f1000research.170512.r336725

**Published:** 2024-11-25

**Authors:** Ganugapanta Vivek Reddy

**Affiliations:** 1 Narayana Dental College, Nellore,, India

Demineralisation around the brackets is a matter of concern ,need to take steps in prevention ,lot of research is going on to reduce it, your efforts to compare gives audience a complete understanding and insight in to material aspects .

 Few clarifications

 1.How was sample size determined per group .

 2. Type of curing Used in the study.

 3.Reasoning behind selection of material.

Is the work clearly and accurately presented and does it cite the current literature?

Yes

If applicable, is the statistical analysis and its interpretation appropriate?

Yes

Are all the source data underlying the results available to ensure full reproducibility?

Yes

Is the study design appropriate and is the work technically sound?

Yes

Are the conclusions drawn adequately supported by the results?

Yes

Are sufficient details of methods and analysis provided to allow replication by others?

Yes

Reviewer Expertise:

cbct

I confirm that I have read this submission and believe that I have an appropriate level of expertise to confirm that it is of an acceptable scientific standard, however I have significant reservations, as outlined above.

# Response

**Published:** 2024-12-04

**Authors:** Nishu Singla

**Affiliations:** Public Health Dentistry, Manipal Academy of Higher Education, Manipal, Karnataka, India

1.How was sample size determined per group .

 Based on a previous study, the sample size of 24 premolar teeth (8 in each group; 3 groups) was determined using power analysis

- **Mean 1 (μ₁) = 2.40800**
- **Mean 2 (μ₂) = 2.40800**
- **Pooled Standard Deviation (SD pooled) = 0.11219**
- **Number of groups (k) = 3**
- **Significance level (α)**: 0.05 
- **Power (1 - β)**: 80% 

Barik AK, Duggal R: Comparative evaluation of fluoride release from chemically cured and light-cured orthodontic bonding agents and surface alteration of enamel: an in vitro study. *J. Indian Orthod. Soc.* 2020 Jul; **54**(3): 233–239.

 2. Type of curing Used in the study.

 The brackets were cured using a 3M S10 ELIPAR Light Curing Unit set at 1200 mW/cm ^2^, with 10 seconds of light exposure from the incisal edge and another 10 seconds from the gingival, mesial, or distal edges.

 3.Reasoning behind selection of material.

 Waldent Orthobond LC and Koden EZ Bond are relatively new materials and lack extensive documentation on fluoride release, recharge, and re-release capabilities, unlike Transbond Plus Color Change.
